# Time-Course Gene Expression Profiling Reveals a Novel Role of Non-Canonical WNT Signaling During Neural Induction

**DOI:** 10.1038/srep32600

**Published:** 2016-09-07

**Authors:** Cindy Tzu-Ling Huang, Yunlong Tao, Jianfeng Lu, Jeffrey R. Jones, Lucas Fowler, Jason P. Weick, Su-Chun Zhang

**Affiliations:** 1 Waisman Center, University of Wisconsin, Madison, WI 53705, USA; 2 Department of Neurosciences, University of New Mexico, Albuquerque, NM 87131, USA; 3 Department of Neuroscience and Department of Neurology, School of Medicine and Public Health, University of Wisconsin, Madison, WI 53705, USA

## Abstract

The process of neuroepithelial differentiation from human pluripotent stem cells (PSCs) resembles *in vivo* neuroectoderm induction in the temporal course, morphogenesis, and biochemical changes. This *in vitro* model is therefore well-suited to reveal previously unknown molecular mechanisms underlying neural induction in humans. By transcriptome analysis of cells along PSC differentiation to early neuroepithelia at day 6 and definitive neuroepithelia at day 10, we found downregulation of genes that are associated with TGF-β and canonical WNT/β-CATENIN signaling, confirming the roles of classical signaling in human neural induction. Interestingly, WNT/Ca^2+^ signaling was upregulated. Pharmacological inhibition of the downstream effector of WNT/Ca^2+^ pathway, Ca^2+^/calmodulin-dependent protein kinase II (CaMKII), led to an inhibition of the neural marker PAX6 and upregulation of epidermal marker K18, suggesting that Ca^2+^/CaMKII signaling promotes neural induction by preventing the alternative epidermal fate. In addition, our analyses revealed known and novel expression patterns of genes that are involved in DNA methylation, histone modification, as well as epithelial-mesenchymal transition, highlighting potential roles of those genes and signaling pathways during neural differentiation.

Neural induction is a process by which the pluripotent inner cell mass becomes restricted to a multipotent neuroectoderm (NE) fate. Evidence from animal studies has suggested that several signaling pathways play essential roles in neural induction. The bone morphogenetic protein (BMP) pathway promotes the epidermal fate and is inhibited in neural tissues by BMP antagonists, including Noggin, Chordin, and Follistatin^1^,^2^,^3^,^4^. The role of WNT signaling pathway in neural induction has been controversial. Studies from chick and *Xenopus* have shown that inhibition of WNT signaling is required for neural induction, while one study suggests that overexpression of WNT ligands promotes the neural fate in *Xenopus*^5^,^6^,^7^. Whether the signaling pathways underlying neural induction that are learned from *Xenopus* and chick are conserved in mammals, including humans, is less well known.

Embryonic stem cells (ESCs), isolated from the inner cell mass of a preimplantation embryo, can differentiate to all cell types of the body, including neural cells^8^. ESCs thus offer a useful model to examine early embryonic development, including neural induction in mammals. The *in vitro* differentiation scheme follows *in vivo* developmental timing; and the cells exhibit typical neural epithelial morphology and have the capability to be patterned by morphogens^9^,^10^. The *in vitro* differentiated cells can ultimately generate functional neurons, astrocytes and oligodendrocytes^11^,^12^,^13^,^14^,^15^,^16^. Studies using the ESC model suggest that many of the signaling pathways learned from lower vertebrates appear to play in mammals. However, it is not known if mammals or primates utilize new signaling pathways or classical pathways but in a different manner for neural induction^17^,^18^,^19^.

Global gene expression profiling followed by advanced bioinformatics analyses enables the identification of signaling pathways that are associated with a developmental process. Microarray profiling of gene expression from a population of cells may be biased by the proportion of a target cell type in a mixture. Hence, pure, or highly enriched cells are desirable. During hESC neural differentiation in the absence of exogenous factors, the relatively uniform ESC population is converted to highly enriched (~90%) neuroepithelia in 10 days, which enables gene expression profiles to reflect the respective cell types. During the transition period, stem cells exit the pluripotent state by down regulating pluripotency transcription factors (TFs), such as *OCT4* and *NANOG* before they acquire a neural fate by expressing early neuroectoderm genes, such as *PAX6*, *SOX1*, *ZEB2*, and *NCAM* around day 6^10^,^20^. Hence, day 6 is a critical stage by which hESCs transition to early neuroepithelia. Molecular profiling of these three stages would reveal dynamic changes in gene expressions and signaling pathways that are associated with the cellular conversion from ESCs to NE.

In this study, we examined the gene expression profiles from pluripotent ESC stage to NE stage to identify genes that may contribute to neural induction. Our study revealed known and novel expression patterns of genes that are involved in forebrain development, DNA methylation, histone modification, as well as epithelial-mesenchymal transition, which are validated with qRT-PCR. These findings highlight potential unique roles of those genes and signaling pathways during neural induction. In particular, pathway analyses revealed upregulation of WNT/Ca^2+^ signaling and pharmacological inhibition of the downstream effector of WNT/Ca^2+^ pathway, Ca^2+^/calmodulin-dependent protein kinase II (CaMKII) resulted in inhibition of the neural but upregulation of epidermal markers, suggesting a role of non-canonical WNT signaling in partitioning the neural vs. epidermal fate during neural induction.

## Results

### The early neuroectoderm expresses predominantly forebrain genes

**Figure 1:**
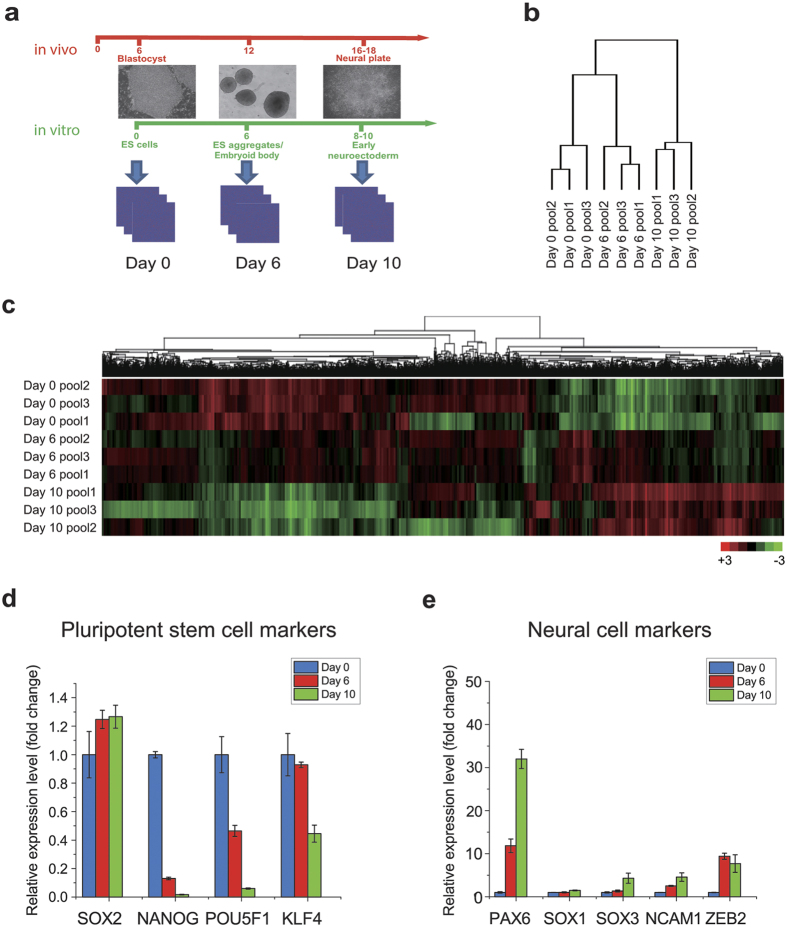
Expression profiling along *in vitro* neural differentiation from hESCs. (**a**) Strategy for microarray analyses. Three time points of neural differentiation are analyzed: day 0 (ESCs), day 6 (ESC aggregates), and day 10 (Early neuroectoderm). (**b**) Hierarchical clustering of three time points using the top 7% of the differentially expressed genes. (**c**) Heatmap of gene expression. Upregulated genes are displayed as red whereas downregulated genes are displayed as green. (**d,e**) Time course expression pattern of known pluripotent stem cell genes (**d**) and neural genes (**e**), using the average of biological replicates for each time point (Mean ± SD).

Human ESCs, under a chemically defined condition, convert to NE cells by day 10, during which day 6 represents a transitional stage when cells are downregulating pluripotent genes and upregulating neural genes^10^,^15^. We therefore collected samples from cells at the three critical time points (day 0, day 6, and day 10) of *in vitro* neural differentiation for microarray analysis (Fig. 1a). Hierarchical clustering and principal component analysis (PCA) showed that the global gene expression at day 10 was most distinct from the other two time points (Fig. 1b and Suppl. Fig. 1). Global gene expression patterns from day 0 and day 10, as shown in the heatmap (Fig. 1c), indicated that core regulatory TF for pluripotency, *POU5F1 (OCT4*), *NANOG* and *KLF4*, are highly expressed at the ESC stage (day 0) and dramatically downregulated upon differentiation (Fig. 1d). Expression of neural genes, including *PAX6*, *SOX1*, *NCAM*, *SOX3*, and *ZEB2*, begins at day 6 and continues to rise over the time course (Fig. 1e). Expression of *SOX2*, a pluripotent as well as a neural TF, does not change over this time course (Fig. 1d). Expression of mesodermal markers (*CD4*, *GATA*, RUNX1 and *T*) and endodermal markers (*GATA1*, *SOX17*, *AFP* and *SOX7*) stay similarly compared to the ESC stage (Suppl. Table 1). Thus, the temporal gene expression pattern corresponds cellular differentiation.

**Figure 2:**
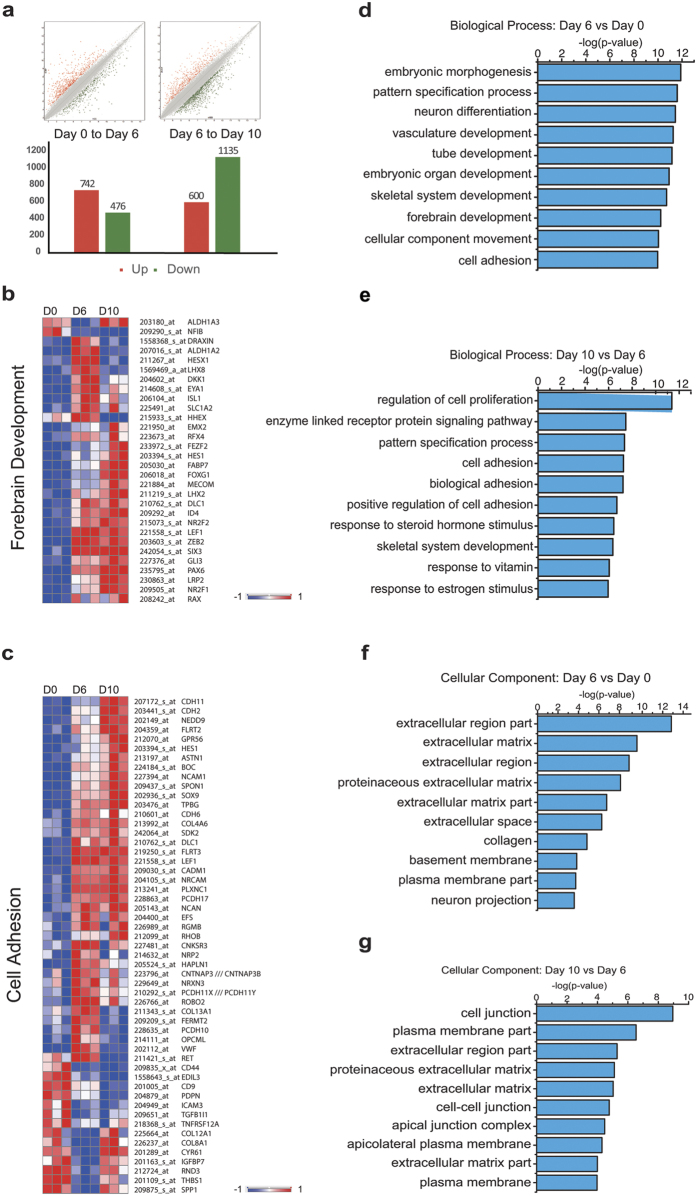
DEGs associated with forebrain development and epithelial-mesenchymal transition during neural differentiation. (**a**) Number of DEGs at each differentiation stage, analyzed by pairwise comparison between the two sequential time points, where the preceding time point is used as the reference group. Two stages (day 0 vs day 6 and day 6 vs day 10) are examined. In upper panels, each red dot represents an upregulated gene, whereas a green dot represents a downregulated gene. (**b**) Heatmap showing the DEGs involved in forebrain development in neural differentiation. (**c**) Heatmap showing the DEGs involved in epithelial-mesenchymal transition in neural differentiation. (**d,e**) Enriched GO terms of biological process of the two differentiation stages. (**f,g**) Enriched GO terms of cellular component of the two stages. The data are presented in –log (p-value) scale.

The differentially expressed genes (DEGs, ≥2 fold) were determined based on the relative expression to the precedent time points (Fig. 2a). Gene Ontology (GO) analysis of the DEGs confirmed the expression of several genes that are predominantly involved in forebrain development, including *PAX6*, *HESX1*, *SIX3*, *RAX*, *LHX2* and *FOXG1* (Fig. 2b,d)^21^,^22^,^23^,^24^,^25^,^26^. Thus, these data are consistent with the default forebrain fate during *in vivo* development and *in vitro* differentiation^10^,^27^,^28^.

Analysis of the top 30 TFs that are up- and down-regulated at day 6 and day 10 of neural differentiation (Suppl. Tables 2 and 3) revealed TFs that are less well known in neural development, including those involved in development of hematopoietic (*BCL11A*, *BCL11B*, and *ZBTB16*), limb (*SP8* and *TFAP2B*), blood vessel/tubular system of lung (*EPAS1*), cardiovascular system (*HEY2*), and hair cell differentiation (*LEF1*). The dynamic expression of these genes was validated using qRT-PCR analysis (Suppl. Fig. 2). Interestingly, *BCL11A* (*CTIP1*) and *BCL11B* (*CTIP2*), which have been shown to be critical for migration of cortical projection neurons and hippocampal neurogenesis, respectively^29^,^30^, are significantly downregulated by 28.5- and 4.4-fold, respectively, by day 10 of differentiation (Suppl. Tables 2 and 3). *HEY2*, a NOTCH target gene regulating maintenance of neural precursors^31^, is also downregulated by 11.5-fold by day 10 (Suppl. Tables 2 and 3). *ZBTB16* (*PLZF*), a neural rosette marker^32^, is upregulated by 24.2-fold by day 10 of differentiation. *SP8*, crucial for closure of neuropore in E8 mouse embryos^33^, is highly upregulated in neural induction (Suppl. Tables 2 and 3). Similar expression pattern of these genes was observed in WA01 hESC-derived NE (Suppl. Fig. 2). These findings suggest the pleiotropic function of these newly identified neural induction-related TFs and highlight a need of further investigation of their roles in neural induction.

### Dynamic epigenetic regulation during neural differentiation

**Figure 3:**
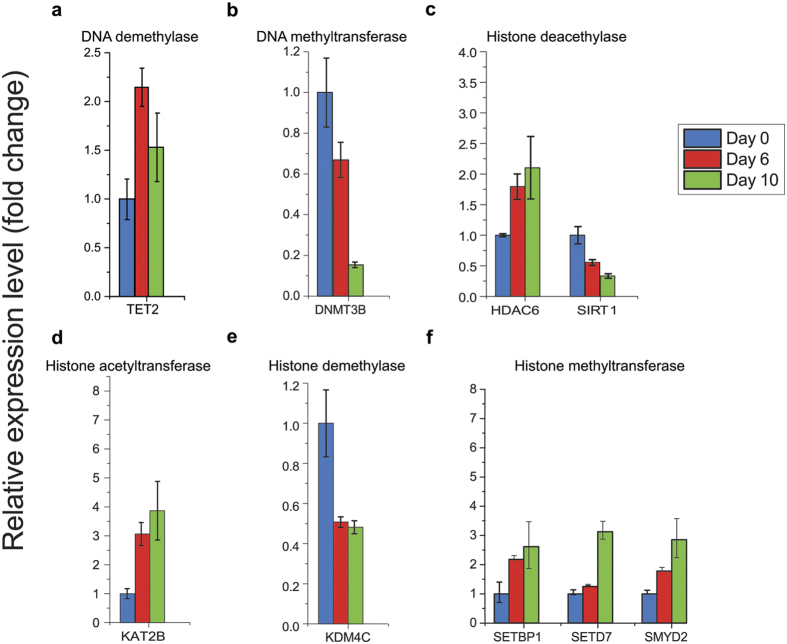
Epigenetic modifiers during neural differentiation. (**a,b**) Time course expression pattern of epigenetic modifiers for DNA de/methylation: DNA demethylase (**a**) and DNA methyltransferase (**b**). (**c,d**) Time course expression pattern of epigenetic modifiers for histone de/acetylation: histone deacetylase (**c**) and histone acetyltransferase (**d**). (**e,f**) Time course expression pattern of epigenetic modifiers for histone de/methylation: histone demethylase (**e**) and histone methyltransferase (**f**).

Little is known about epigenetic regulation of neural induction. Here, we analyzed the expression pattern of genes that are involved in epigenetic regulation (Fig. 3 and Suppl. Fig. 3). We found that expression of *DNMT3B*, one of the DNA methyltransferases for de novo methylation, is downregulated by 6.5-fold at day 10 compared to day 0 (Fig. 3b and Suppl. Fig. 2). In contrast, expression of *TET2*, a methylcytosine dioxygenase that is responsible for catalyzing the conversion of 5 mC to 5 hmC, resulting in DNA de-methylation, is upregulated during neural differentiation (Fig. 3a and Suppl. Fig. 2).

Histone acetylation and methylation regulate access of TFs to chromatin^34^. Our analysis revealed a complex picture of histone regulator expression. For instance, the histone acetyltransferase *KAT2B* (*PCAF*) was upregulated 3.9-fold at day 10 (Fig. 3d and Suppl. Fig. 2), while the histone deacetylases, *SIRT1* and *HDAC6*, were moderately downregulated by 3.0-fold and upregulated by 2.1-fold, respectively, by day 10 of neural differentiation (Fig. 3c and Suppl. Fig. 2).

Histone methyltransferases can be divided into two types: lysine specific and arginine specific modifications. The lysine specific methyltransferases can be further subdivided into two groups by the structure of a catalytic SET domain. Among the SET domain histone methyltransferases, *SETBP1* and *SETD7* expression was found to be gradually upregulated during neural differentiation, reaching a ~3-fold increase by day 10 (Fig. 3f and Suppl. Fig. 2). *SMYD2* was also found moderately upregulated by 2.9-fold by day 10 (Fig. 3f and Suppl. Fig. 2). In addition, expression of *KDM4C*, a histone demethylases, was downregulated by 2.1-fold when cells reached day 6 of differentiation (Fig. 3e and Suppl. Fig. 2). Taken together, the dynamic expression of several DNA and histone modifiers suggests a regulatory network governing gene regulation during neural differentiation.

### Epithelial-mesenchymal transition (EMT) during neural induction

During early embryogenesis, the inner cell mass undergoes extensive movement in the formation of three germ layers. The three stages of cells from hESCs to NE provide a platform to look at genes that are expressed in the sequential steps. GO analysis of DEGs revealed activation/repression of genes of extracellular matrix components, such as *NCAM1*, *CADHERIN* family (*CDH11*, *CDH2*, and *CDH6*) and *COLLAGEN* family (*COL4A6*, *COL13A*, *COL12A1*, and *COL8A1*) during the transition of hESCs to NE (Fig. 2c,e-g). Particularly, we observed that *CDH1* (*E-CADHERIN*) is downregulated 9.5-fold while *CDH2* (*N-CADHERIN*) is upregulated 6.1-fold, highly consistent with previous studies^35^. In addition, ZEB1, a TF that promotes EMT^36^, was dramatically increased by 23.8-fold from day 0 to day 10 (Table 1). Several epithelial-specific genes that are associated with EMT are regulated by ZEB1 (Table 1)^37^. Expression of *ZEB1*-repressed genes that are associated with tight junction (*OCLN*, *CLDN7*, *F11R*, and *MARVELD2*), cell polarity (*CRB3*), desmosomes/epidermis (*PPL*, *DSG2*, *EPPK1*, *SH3YL1*, and *DMKN*), cell surface receptor (*CD24*), and vesicle transport (*TMEM30B* and *MAL2*) are consistently downregulated during neural differentiation. Another transcriptional factor, SNAI2 that is known to cooperate with SNAI1 to repress epithelial genes, was also upregulated during differentiation (Table 1)^38^. Together, our profiling analysis suggests a potential role of EMT in neural induction.

### Dynamics of extracellular signaling pathways in neural induction

**Figure 4:**
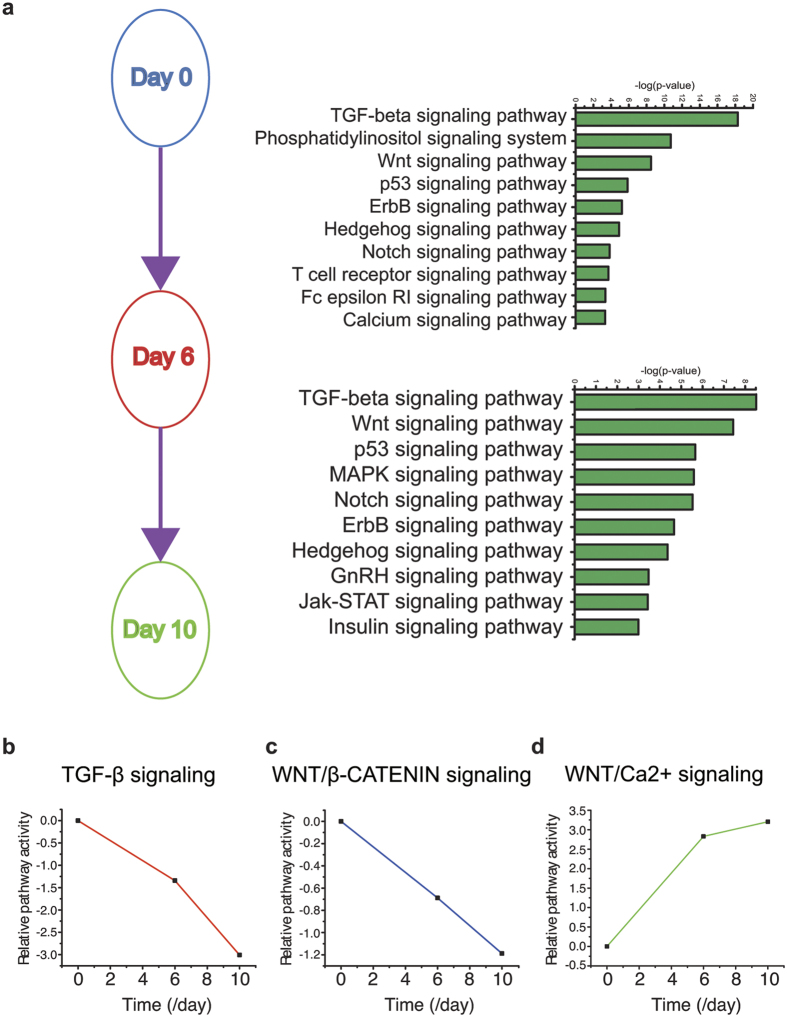
Change of signal transduction during neural induction. (**a**) Top 10 pathways that are statistically significant from day 0 to day 6 and day 6 to day 10. (**b–d**) Pathway activity of TGF-β signaling (b), WNT/β-CATENIN signaling (**c**), and WNT/Ca^2+^ signaling (**d**) from day 0 to day 10.

Several extracellular signaling pathways, such as TGF-β and WNT signaling during neural induction, are well established in a number of model systems^39^. Using Onto-Pathway Express and IPA pathway analyses we asked which pathway takes place during neural induction in our system. We identified enrichment of TGF-β signaling pathway (Fig. 4a,b), specifically two ligands INHBA (subunits of ACTIVIN A) and NODAL, which were downregulated by at least 2–3 fold during neural differentiation. The expression of *SMAD2*, an R-SMAD of TGF-β signaling stays constant, although *SMAD3* is upregulated by 2.8-fold at day 10. Interestingly, expression of *SMAD7*, an I-SMAD that competes with *SMAD2* and *SMAD3* for SMAD4 binding, was downregulated during neural differentiation (Table 2).

Inhibition of the BMP subfamily of TGF-β signaling is required for neural induction^39^,^40^. Consistently, we found multiple levels of BMP inhibition. While a host of BMP ligands and several receptors exist, only *BMPR1B* was found significantly upregulated at day 10 (6.4-fold; Table 2), consistent with localized expression in early rodent anterior telencephalon^41^. ZEB2, a TF that binds to SMAD to inhibit BMP signaling is highly upregulated (8- to 10 fold) during neural induction. Moreover, expression of the BMP antagonists, *NOGGIN*, *GREMLIN*, and *FOLLISTATIN*, were significantly upregulated during differentiation (Table 2), consistent with the inhibition of TGF-β activity during differentiation (Fig. 4b).

The role of WNT pathway in neural induction is controversial^5^,^6^,^7^. From our pathway activity analysis, canonical WNT in which *β-CATENIN* (*CTNNB1*) acts as the effector was shown to be decreased during neural induction whereas the non-canonical WNT/calcium (Ca^2+^) activity was increased (Fig. 4a,c,d). Most *WNT* ligands were not significantly changed, owing to the high level of basal expression of the *WNT* ligands in the ESCs (Table 2). In contrast, expression of the WNT antagonist *DICKKOPF 1* (*DKK1*) was significantly increased at day 6 and day 10 (Table 2), suggesting that WNT signaling is being inhibited, consistent with downregulation of canonical WNT/β-CAT activity (Fig. 4c).

Interestingly, *WNT5B*, normally associated with the non-canonical WNT pathways, such as WNT-ROR2 and WNT/Ca^2+^ pathways^42^,^43^,^44^, is increased during neural differentiation. *ROR2*, a receptor protein tyrosine kinase associated with the non-canonical WNT pathway, is upregulated during neural differentiation (Table 2). ROR2 interacts with WNT5B to enhance cell migration^42^, which is important during neural differentiation. Taken together, these analyses suggest that the non-canonical WNT5B pathway may be important for neural differentiation.

**Figure 5:**
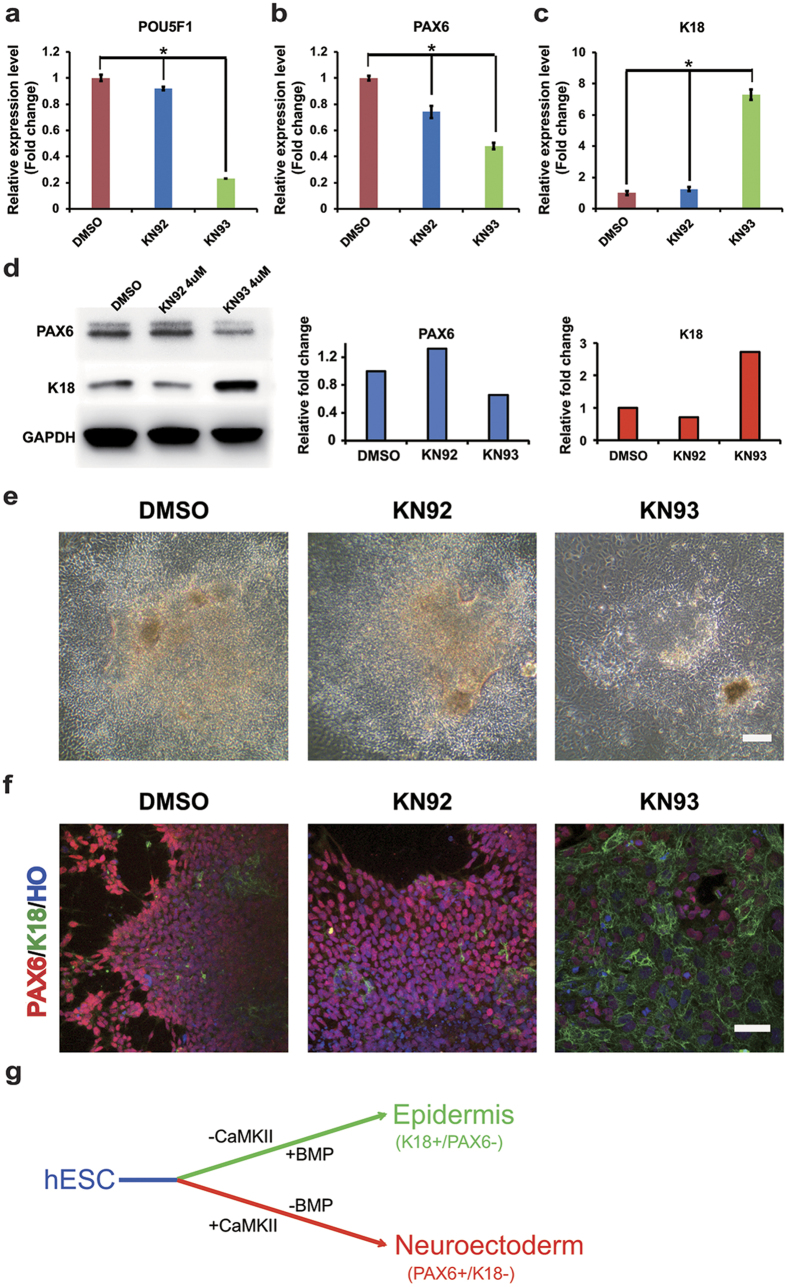
Ca^2+^/CaMKII phosphorylating activity is required for neural differentiation from hESCs. (**a–c**) qRT-PCR analysis of (**a**) *POU5F1*, (**b**) *PAX6*, and (**c**) *K18* in DMSO-, KN92- and KN93-treated neural differentiating cells. *p < 0.05 in comparison with the value from control cells. (**d**) Western blotting analysis and its quantification of PAX6 and K18 in DMSO-, KN92- and KN93-treated neural differentiating cells. (**e**) Immunostaining of PAX6 (red) and K18 (green) at day 8 of differentiated cells after treatment of DMSO, KN92, and KN93 from day 2 to day 7. Scale bar = 50 um. (**f**) Brightfield of differentiated cells that were treated with DMSO, KN92, or KN93. Scale bar = 10um. (**g**) A model signaling pathways involved in neural induction from hESCs.

To test our hypothesis, we examined whether manipulation of Ca^2+^/calmodulin-dependent protein kinase II (CaMKII), a downstream kinase that is regulated by WNT/Ca^2+^ signaling, affects the propensity of acquiring the neural fate. We examined the level of the neural marker PAX6 in response to treatment with KN93, a pharmacological inhibitor of CaMKII phosphorylating activity, which is activated by an increase of Ca^2+^. After exposure to KN93 (4 µM) from day 2 to day 6, the expression of PAX6 was significantly reduced as compared to cells treated with KN92 (inactive form of KN93) (Fig. 5b,c), suggesting that CaMKII phosphorylating activity is required for neural induction. Interestingly, levels of the pluripotency marker POU5F1 were also reduced (Fig. 5a), suggesting that the inhibition of neural differentiation is not due to prevention of ESC differentiation. WNT signaling in general is important in regulating the boundary between the epidermal and neural ectoderm although the exact signaling is not known. We observed cells that were treated with KN93 exhibited a reduced area of columnar rosette cells but a large area of flat epithelial cells in the colony whereas the control groups (DMSO or KN92 treatments) displayed rosette neuroepithelial morphology in the colony (Fig. 5e), indicating the repression of neural induction by KN93 treatment. Interestingly, the expression level of another early neural marker gene *ZFP521*^18^, which promotes the conversion of mouse ESCs to neural progenitors through the inhibition of BMP signaling, was similar between the control groups (DMSO and KN92) and KN93-treated group (Suppl. Fig. 4), suggesting that CaMKII/Ca^2+^ signaling may regulate a subset of neural genes, such as *PAX6* gene, directly or indirectly. Moreover, the forebrain marker *FOXG1* was significantly higher in the KN93 group (Suppl. Fig. 4), suggesting that CaMKII/Ca^2+^ pathway may also play a role in forebrain patterning. By examining the neural (PAX6) and epidermal (K18) marker expression, we further observed that the KN93-treated cells preferentially differentiated to the epidermal fate (Fig. 5d,f). This suggests that the CaMKII/Ca^2+^ pathway regulates the choice between the neural and epidermal fates during differentiation from hESCs (Fig. 5g). Our findings demonstrate that activation of WNT/Ca^2+^ pathway is crucial to neural induction, possibly through regulation of CaMKII phosphorylation.

## Discussion

Our time-course microarray along hESC neural differentiation enables us to interrogate the dynamics of global gene expression and signaling pathways that are involved in early neural differentiation. Indeed, the gene expression profiles from sequential differentiation stages confirm the conversion of ESCs to NE, as indicated by downregulation of pluripotent genes and upregulation of forebrain NE genes. Gene ontology analysis reveals potential roles of less well-studied pathways in neural induction, including EMT, as indicated by specific expression patterns of extracellular matrix protein and adhesion genes during the transition period, and epigenetic regulation, as indicated by a characteristic expression pattern of genes that are involved in DNA methylation and histone modification. Pathway analysis confirms the roles of classical TGF-β and canonical WNT/β-CATENIN signaling in human neural induction. Importantly, it also suggests a role of non-canonical WNT/Ca^2+^ signaling in partitioning the epidermal vs NE fate during human neural induction, which we have now confirmed by intervening the pathway.

EMT is a common phenomenon during embryonic development; it is nevertheless rarely mentioned during neural induction. Results from DEG and GO analyses suggest that EMT may play a role in neural induction. This is evidenced by upregulation of ZEB1, a central regulator for EMT, along differentiation and concomitant downregulation of epithelial-specific genes that are repressed by ZEB1. It is also suggested by the upregulation of the SNAIL family genes, especially SNAI2. It should be noted that such changes may also be attributed to neural crest differentiation, which cannot be excluded from our neural differentiation cultures. Together, our analysis suggests a role of EMT during human neural induction.

Epigenetic regulation has gained increasing attention during embryonic development^45^,^46^ although its function in neural development remains unclear. Our finding that *DNMT3B* is significantly downregulated during hESC neural induction suggests that modification of neural developmental genes at the DNA level plays a role in neural induction. Recently, TET2 is shown to be recruited to neurodevelopmental gene loci for methylcytosine hydroxylation^47^. Another layer of gene regulation is histone modification. Histone acetylation on lysine leads to activation of its target genes. During hESC differentiation, the histone acetylase, KAT2B is upregulated. In *Xenopus*, KAT2B interacts with SIP1 to repress BMP signaling^48^. Hence, upregulation of KAT2B is consistent with the acquisition of the neural fate. Suppression of SIRT1 has been shown to promote neural differentiation of mouse induced pluripotent stem cells to neural stem cells^49^. In mouse ESCs, HDAC6 has been shown to regulate TIP60 (KAT5)-P400 target genes through interaction with TIP60-P400 complex, which activates genes required for proliferation and silences genes that promote differentiation^50^. In contrast, histone methylation can activate or repress gene expression depending on the position and numbers of methyl groups added to the lysine residue. The histone methyltransferases, SETBP1 SETD7 and SMYD2, are upregulated during neural differentiation; however, their roles in neural induction are not well-understood. Altogether, it is now of interest to investigate the roles of these epigenetic modifiers during neural induction.

Signaling pathway analysis allows us to gain insights into the molecular basis of neural differentiation. Indeed, the activity of TGF-β signaling and WNT/CATENIN signaling is decreased along neural differentiation, consistent with findings learned from other model systems^40^. This is evidenced by highly upregulated expression of BMP inhibitors NOGGIN, GREMLIN, and FOLLISTATIN even though the expression of BMP ligands and most SMAD genes are not changed. It is noteworthy to mention that TGF-β signaling through SMAD is associated with EMT, which may explain why most SMAD proteins are retained during neural differentiation even though TGF-β pathway should be inhibited during neural induction^38^.

One caveat of pathway analysis is that the analysis does not indicate whether a signaling is causal or secondary and its interpretation is often complicated by the crosstalk between pathways. Indeed, our pathway activity analysis showed downregulation of WNT/β-CATENIN activity but also upregulation of some of the WNT ligands, WNT5B and WNT8 during neural differentiation. Given that WNT5B is associated with non-canonical WNT pathways, such as WNT-ROR2 and WNT/Ca^2+^ pathways^42^,^43^,^44^, we speculate that WNT5B or WNT8 is responsible for the upregulation of the non-canonical WNT pathway. Indeed, *ROR2*, a receptor protein tyrosine kinase associated with the non-canonical WNT pathway, is upregulated along neural differentiation. Our analysis with the CaMKII inhibitor KN93 further confirms that the non-canonical WNT signaling promotes the neural but inhibits the epidermal fate without blocking hESC differentiation. In addition, WNT5B has been implicated in partially inhibiting the canonical WNT/β-CATENIN signaling pathway^51^, which may lead to a decrease of the WNT/β-CATENIN pathway during neural induction. Together, we propose that inhibition of canonical WNT signaling promotes hESC differentiation toward the (neural) ectodermal fate and activation of the non-canonical WNT signaling enhances the neural fate by limiting the epidermal choice.

Our analysis on gene expression profiles during sequential steps of neural differentiation validates the genes and signaling pathways that are known to be essential for neural induction, highlighting the utility of the hESC differentiation platform to better understand early human embryonic neural differentiation. Importantly, it also reveals novel genes and signaling pathways, such as EMT, epigenetic regulation, and non-canonical WNT signaling, that play potential roles during human neural induction. This information will hopefully rekindle the interest in the fundamental process of neural induction.

## Methods

### Cell Culture

WA09 and WA01 hESCs were maintained on a feeder layer of mouse embryonic fibroblasts^8^. Neural differentiation was initiated by detaching hESCs from feeder cells, and then the cells were suspended in the hESC medium (DMEM/F12, 20% knockout replacement serum, nonessential amino acids, 2 mM glutamine, and 100 μM β-mercaptoethanol) for 4 days. The ESC aggregates were then suspended in neural medium (DMEM/F12, N2 supplement, nonessential amino acids, and 2 μg/ml heparin) for 2 days and attached to laminin-coated plates. At around day 8–10, the primitive columnar neuroepithelial cells appeared and were organized into rosette structures.

### Microarray sample Preparation and Microarray analyses

The differentiation was repeated three times to obtain biological replicates. Gene expression profiles were performed using Affymetrix HG-U133 Plus 2.0 Genechip microarrays that contain 54, 613 probesets (Affymetrix, Santa Clara, CA). For each experimental group (day 0, day 6, and day 10), three biological replicates were hybridized. Dataset quality was assessed by Affymetrix quality control metrics as well as by principal component analysis (PCA). All the microarray data were normalized by Affymetrix Expression Console software using the RMA algorithm. Normalized expression data were then compared and analyzed by Affymetrix Transcriptome Analysis Console 2.0 (TAC 2.0). The array data were deposited at the NIH Neuroscience Microarray Consortium and in the ArrayExpress database (accession number E-MEXP-2426).

Each development stage during neural differentiation was analyzed by pairwise comparison (day 6 versus day 0, day 10 versus day 6). Genes with a ≥2-fold change and a one-way ANOVA p-value < 0.05 were identified as differentially expressed genes (DEGs). Principal component analysis and hierarchical clustering were analyzed by Partek^®^ Genomics Suite™ software. Gene annotation analysis was performed with Database for Annotation, Visualization and Integrated Discovery (DAVID) using the probe IDs of the DEG lists (http://david.abcc.ncifcrf.gov/summary.jsp). Differentially expressed genes in selected GO terms were chosen to make the gene expression heat map by Gene-E (http://www.broadinstitute.org/cancer/software/GENE-E/). Top 30 transcription factors were screened out based on the GO:0006355 (regulation of transcription, DNA-dependent) or GO 0003700 (transcription factor activity) from the differentially expressed gene (DEG) list. Genes belonging to a specific epigenetic modifying classification (DNA de/methylation, histone de/methylation, and histone de/acetylation) were selected to analyze their expression pattern during neural differentiation to screen for potential epigenetic modifiers that are critical for neural fate determination. Pathway analyses were performed by Pathway-Express from Onto-Tools online website (http://vortex.cs.wayne.edu/projects.htm). For each stage (day 0-day 6, day 6-day 10), differentially expressed genes and fold change values were analyzed. Pathways were ranked by the impact factor based on the gamma p-value. Signal transduction pathway activities were predicted by Ingenuity Pathway analysis (IPA) from QIAGENE (http://www.ingenuity.com/products/ipa). Relative pathway activities were quantified by using the z-score provided by IPA, and the pathway activity in day 0 was defined as 0. Pathway activities in other time points were generated by adding the relative z-score from the previous time points.

### Quantitative Real-Time polymerase chain reaction (qRT-PCR), western blotting and immunocytochemistry

Total RNA was isolated using RNeasy mini kit (Qiagen) according to manufacturer’s manual. One mg of total RNA was used for reverse transcription using iScript cDNA synthesis kit (Bio-Rad). qRT-PCR was performed using iTag Universal Probes Supermix (Bio-Rad) on StepOnePlus (Applied Biosciences). Primer sequences are listed in Suppl. Table 4. Both western blotting (WB) and immunostaining was performed as described previously^17^. Briefly, cells for western blotting were lysed in a lysis buffer containing 1% Nonidet P-40, 50 mM Tris-HCl, pH 8.0, 0.5% sodium deoxycholate, 150 mM NaCl, 5 mM EDTA, and 1X protease inhibitor cocktail (Sigma). Fifteen μg of proteins in the supernatant were boiled in SDS–PAGE sample buffer and separated by SDS–PAGE. The quantification analysis of WB was done using ImageJ. Brightfield images were taken using AmScope MD900E camera and OLYMPUS CKX41 microscope. Cells for immunostaining were fixed in 4% buffered paraformaldehyde (PFA), pH7.4 for 15 min at room temperature. Antibodies that are used: GAPDH (1:3000, Thermo), PAX6 (1:10000 for WB, DSHB; 1:1000 for staining, Covance), and K18 (1:800 for WB, 1:200 for staining, Millipore). The nuclei are stained with Hoechst. Images were collected with a Nikon C1 laser-scanning confocal microscope.

## Additional Information

**How to cite this article**: Huang, C. T.-L. *et al*. Time-Course Gene Expression Profiling Reveals a Novel Role of Non-Canonical WNT Signaling During Neural Induction. *Sci. Rep*. **6**, 32600; doi: 10.1038/srep32600 (2016).

## Supplementary Material

Supplementary Information

## Figures and Tables

**Table 1:** Gene expression changes in EMT process of differentiating ESCs compared to ESCs.

Gene symbol	Day 6	Day 10	Gene symbol	Day 6	Day 10	Gene symbol	Day 6	Day 10
CDH1	−1.3	−9.5	TWIST1	−1.7	−1.5	SNAI1	1	−1.1
CDH2	2.4	6.1	TWIST2	1.2	1.1			
ZEB1	3.1	23.8	SNAI2	4.7	4.5			
**Epithelial-specific genes known to be repressed by ZEB1**								
**Tight junction**			**Desmosomes and epidermis**			**Cell surface receptors**		
OCLN	−1.2	−1.8	PPL	−1.2	−4.6	CD24	−1.5	−4.8
CLDN7	−2.1	−6.6	DSG2	−1	−2.5	**Vesicle transport**		
F11R	1.3	−1.7	EPPK1	1.1	−2.9	TMEM30B	−1.8	−11.4
MARVELD2	1	−2.3	SH3YL1	−1.5	−2.4	MAL2	−1.6	−6.7
**Cell polarity**			DMKN	−1.3	−3.9			
CRB3	−1.1	−1.6						

A positive value means the fold change of upregulation of the gene at Day 6 or 10 compared to ESCs (Day 0). A negative value means the fold change of downregulation of the gene at Day 6 or 10 compared to ESCs (Day 0).

**Table 2:** Gene expression changes in TGF-β, BMP and WNT pathways of differentiating ESCs.

Gene symbol	Day 6	Day 10	Gene symbol	Day 6	Day 10	Gene symbol	Day 6	Day 10
**TGF-β Signaling**			SMAD5	1.4	1.5	FZD3	2.4	4.2
INHBA	−2.5	−2	SMAD6	1.8	1.1	FZD4	1.1	0.9
NODAL	−2.3	−2.8	SMAD7	0.6	0.3	FZD5	2.2	2.7
TGFB1	−1.2	−1.1	SMAD9	1.2	1.3	FZD6	0.8	1
TGFB2	1.3	1.6	ZEB2	9.9	8.1	FZD7	1	0.5
TGFB3	1.1	1.1	NOG	1.7	2.9	FZD8	2.7	3.5
ACVR1B	1.2	−1.3	GREM1	2	2.9	FZD9	0.9	0.9
ACVR1C	−1.4	−1.6	GREM2	1.1	1.1	FZD10	2.6	2
ACVR2B	1.4	1.1	FST	2.7	0.9	LRP1	1.2	1
**BMP Signaling**			**WNT Signaling**			LRP1B	1.1	1
BMP1	1	1.2	WNT1	1.3	1.2	LRP3	1.2	1.2
BMP2	1.6	0.6	WNT2	0.9	1.1	LRP4	1.5	1.4
BMP3	0.9	1	WNT2B	1.1	1.3	LRP5	0.9	1
BMP4	1.3	1.2	WNT3	0.8	1.1	LRP6	1	1.4
BMP5	2.2	1.3	WNT4	1.4	0.9	LRP11	0.5	0.4
BMP6	0.9	0.9	WNT5A	1	1.1	LRPAP1	1.3	1
BMP7	1	1	WNT5B	3.2	21.2	LRP12	1.5	2.3
BMP8A	1	1	WNT6	1	1.3	CTNNB1	0.8	0.9
BMP8B	1	1	WNT7A	1.1	1.3	GSK3B	1.1	0.9
BMP10	0.9	1	WNT7B	1	1.7	ROR1	0.8	0.3
BMP15	0.9	1.2	WNT8B	1.2	1.2	ROR2	5.4	3.7
BMPR1A	0.7	0.6	WNT9A	1	1.1	RYK	1	1.1
BMPR1B	1.2	6.4	WNT10A	0.9	0.9	DKK1	5.5	2.1
BMPR2	0.8	1.1	WNT10B	0.8	0.9	DKK2	1	1.2
SMAD1	1.2	1.4	WNT11	1.1	1.2	DKK3	0.9	0.9
SMAD2	1.3	0.9	WNT16	1	1	DKK4	1	1.1
SMAD3	1.2	2.8	FZD1	3.6	8.1			
SMAD4	1.1	1	FZD2	4.5	4.5			

A positive value means the fold change of upregulation of the gene at Day 6 or 10 compared to ESCs (Day 0). A negative value means the fold change of downregulation of the gene at Day 6 or 10 compared to ESCs (Day 0).
