# Supplementary material for: Time-Course Gene Expression Profiling Reveals a Novel Role of Non-Canonical WNT Signaling During Neural Induction
Source: Sci Rep. 2016 Sep 7;6:32600. doi: 10.1038/srep32600 (PMC5013468; doi:10.1038/srep32600)
Supplement: Supplementary Information [file srep32600-s1.pdf]

Supplementary Information for

Time-Course Gene Expression Profiling Reveals a Novel Role of Non-Canonical WNT Signaling During Neural Induction

Cindy Tzu-Ling Huang[1,4], Yunlong Tao[1,4], Jianfeng Lu[1], Jeffrey R Jones[1], Lucas Fowler[1], Jason P Weick[2], Su-Chun Zhang[1,3,*]

[1]Waisman Center, University of Wisconsin, Madison, WI 53705, USA

[2]Department of Neurosciences, University of New Mexico, Albuquerque, NM 87131

[3]Department of Neuroscience and Department of Neurology, School of Medicine and Public Health, University of Wisconsin, Madison, WI 53705, USA

[4]These authors contributed equally to this work

[*]Correspondence: suchun.zhang@wisc.edu

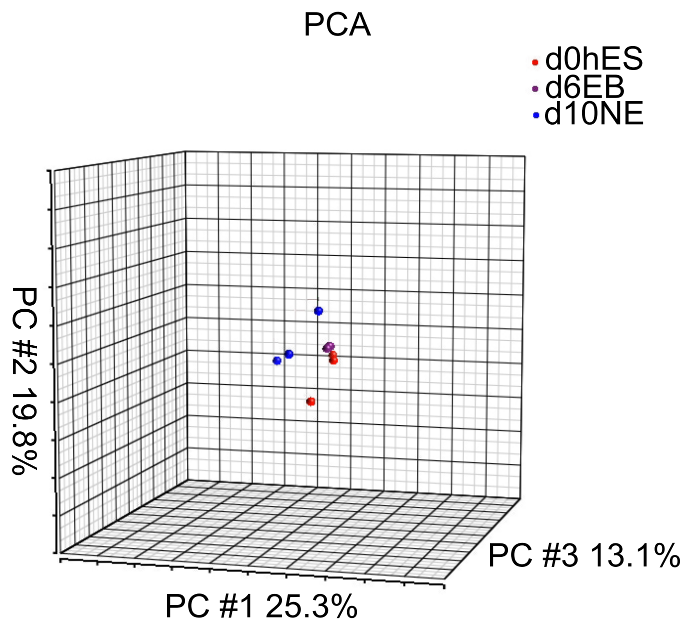

PCA
d0hES
d6EB
d10NE
PC #2 19.8%
PC #3 13.1%
PC #1 25.3%

Suppl. Figure 1. Quality of array data by PCA analysis

Samples from the three time points (day 0, day 6, and day 10) are analyzed. Every time point has three biological replicates. Each dot represents one data set.

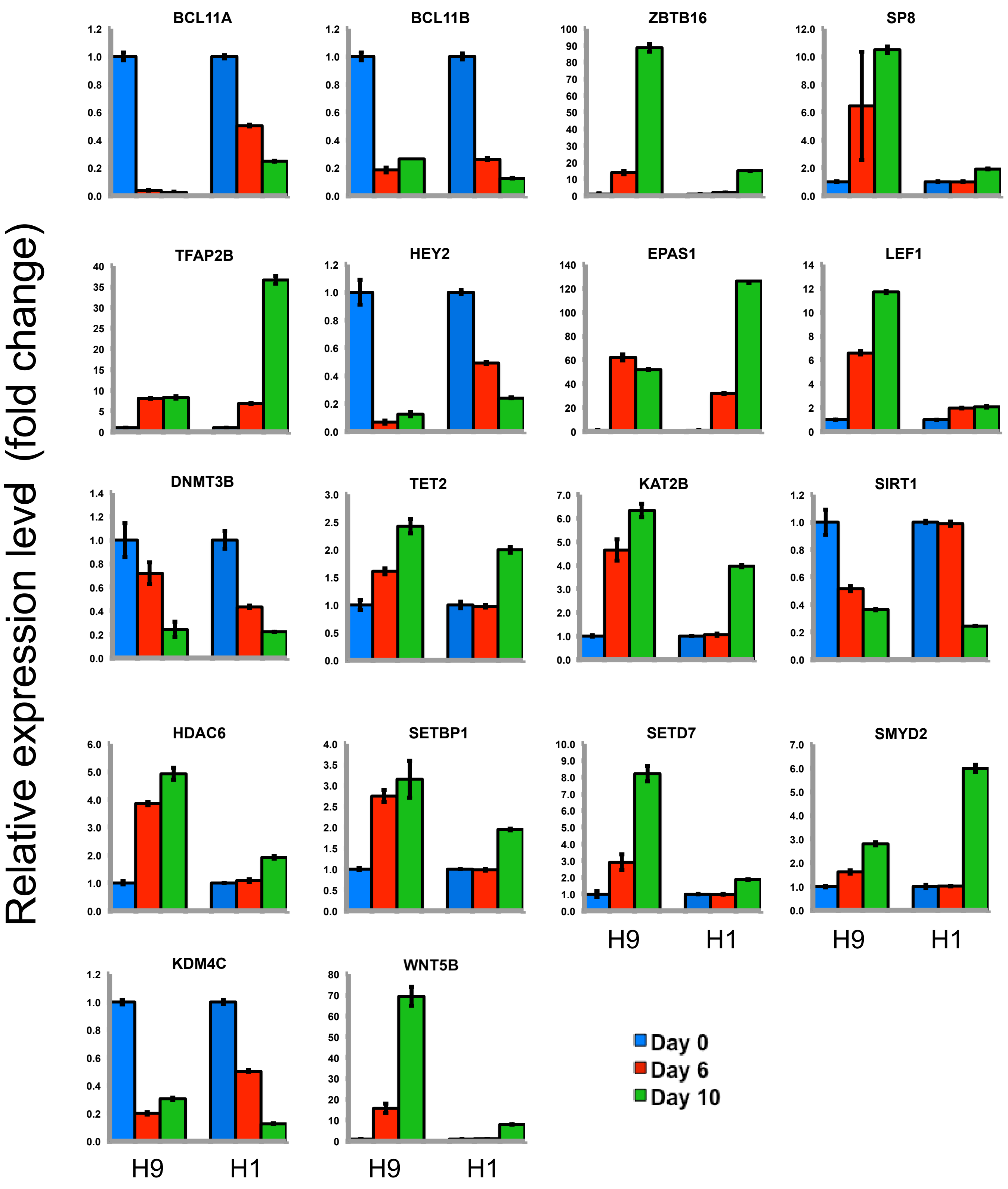
Relative expression level (fold change)
BCL11A
BCL11B
ZBTB16
SP8
TFAP2B
HEY2
EPAS1
LEF1
DNMT3B
TET2
KAT2B
SIRT1
HDAC6
SETBP1
SETD7
SMYD2
KDM4C
WNT5B
H9
H1
Day 0
Day 6
Day 10

Suppl. Figure 2. Time course expression pattern of transcription factors and epigenetic modifiers during neural differentiation from WA09 and WA01 ESCs by qRT-PCR

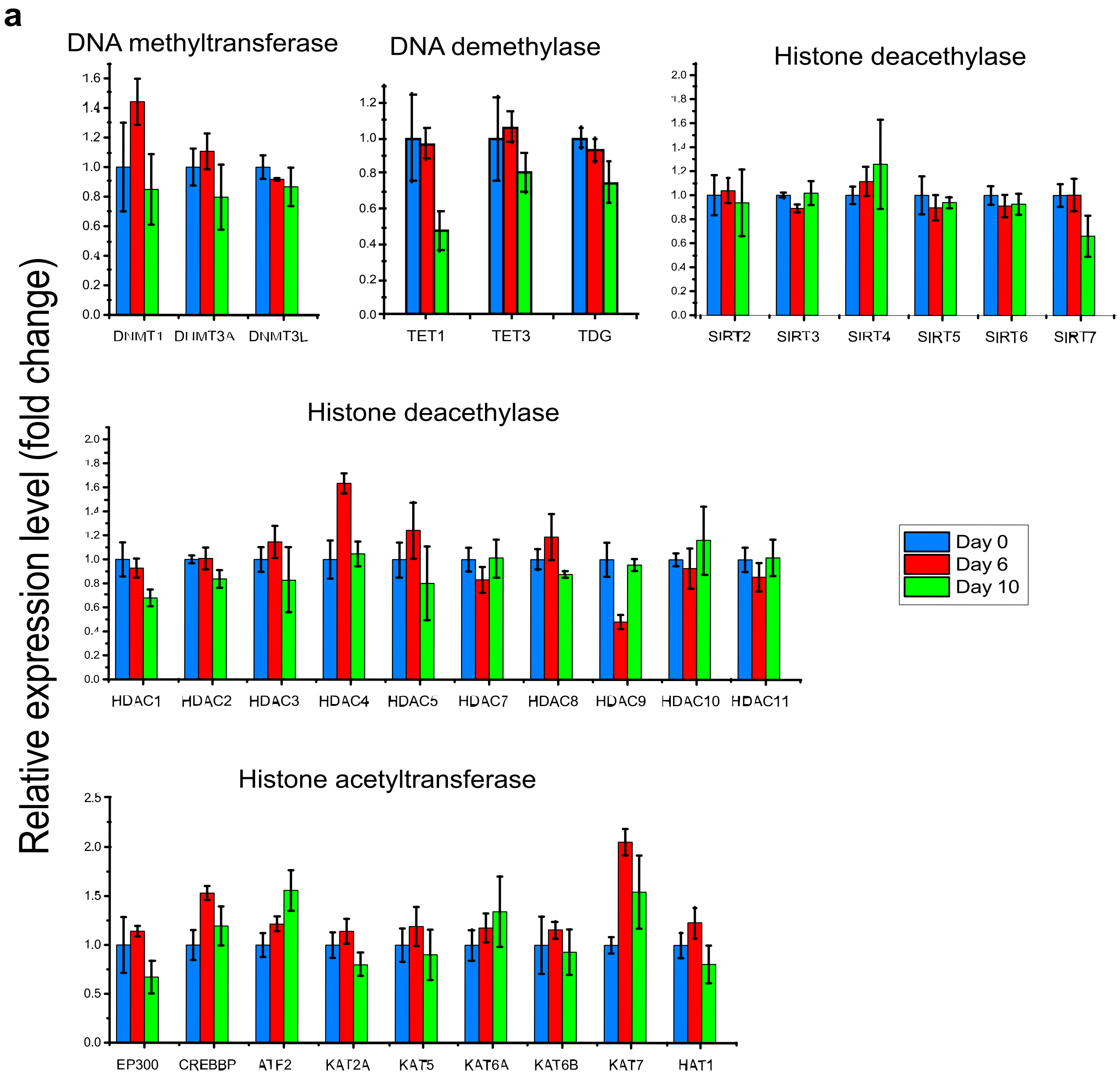

a
Relative expression level (fold change)
DNA methyltransferase
DNMT1
DNMT3A
DNMT3L
DNA demethylase
TET1
TET3
TDG
Histone deacethylase
SIRT2
SIRT3
SIRT4
SIRT5
SIRT6
SIRT7
Histone deacethylase
HDAC1
HDAC2
HDAC3
HDAC4
HDAC5
HDAC7
HDAC8
HDAC9
HDAC10
HDAC11
Day 0
Day 6
Day 10
Histone acetyltransferase
EP300
CREBBP
ATF2
KAT2A
KAT5
KAT6A
KAT6B
KAT7
HAT1

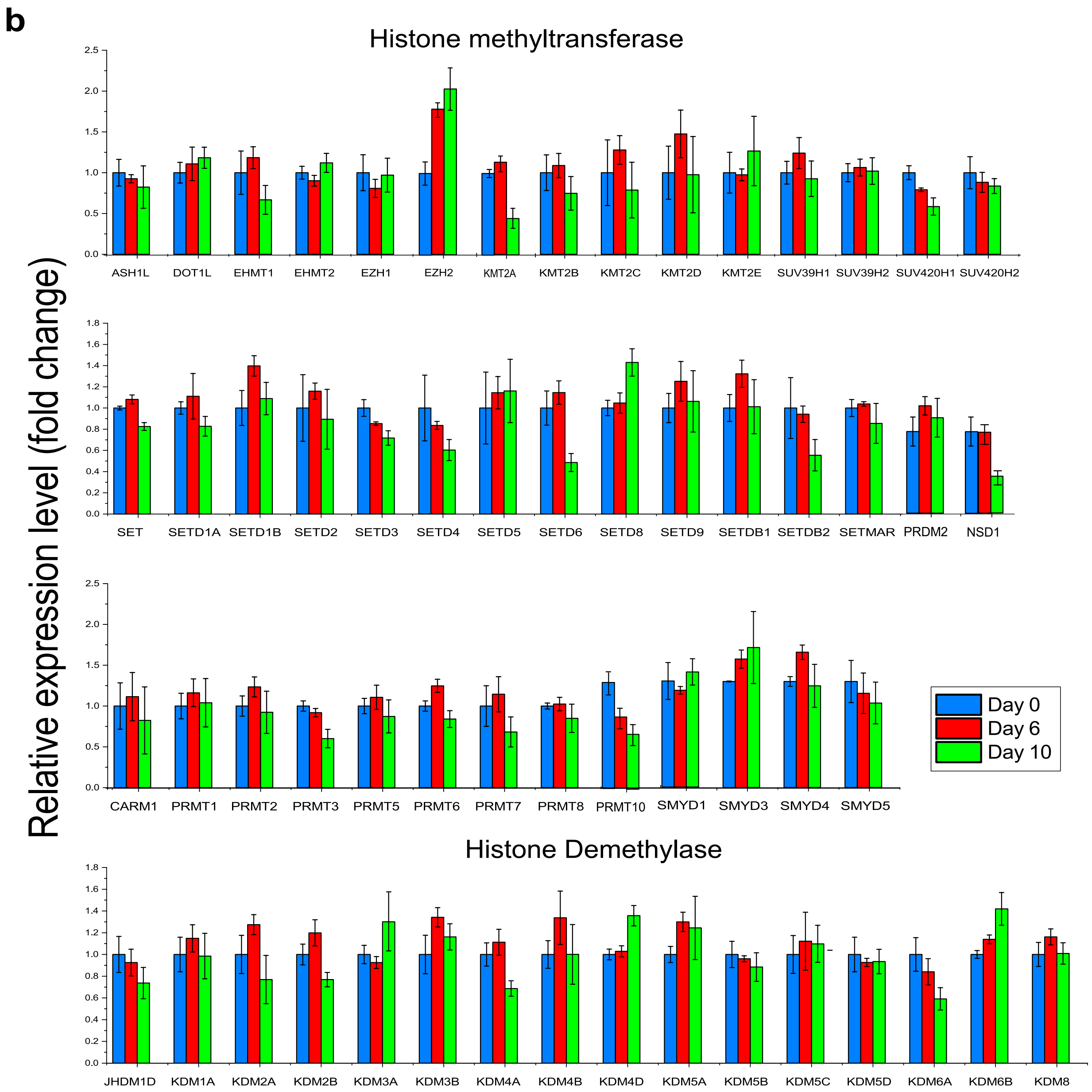
b
Histone methyltransferase
Relative expression level (fold change)
ASH1L
DOT1L
EHMT1
EHMT2
EZH1
EZH2
KMT2A
KMT2B
KMT2C
KMT2D
KMT2E
SUV39H1
SUV39H2
SUV420H1
SUV420H2
SET
SETD1A
SETD1B
SETD2
SETD3
SETD4
SETD5
SETD6
SETD8
SETD9
SETDB1
SETDB2
SETMAR
PRDM2
NSD1
CARM1
PRMT1
PRMT2
PRMT3
PRMT5
PRMT6
PRMT7
PRMT8
PRMT10
SMYD1
SMYD3
SMYD4
SMYD5
Day 0
Day 6
Day 10
Histone Demethylase
JHDM1D
KDM1A
KDM2A
KDM2B
KDM3A
KDM3B
KDM4A
KDM4B
KDM4D
KDM5A
KDM5B
KDM5C
KDM5D
KDM6A
KDM6B
KDM8

Suppl. Figure 3. Time course expression pattern of epigenetic modifiers during neural differentiation

(A) Expression pattern of DNA demethylases, DNA methyltransferases, histone deacetylases, histone acetyltransferases. (B) Expression pattern of histone demethylases, and histone methyltransferases.

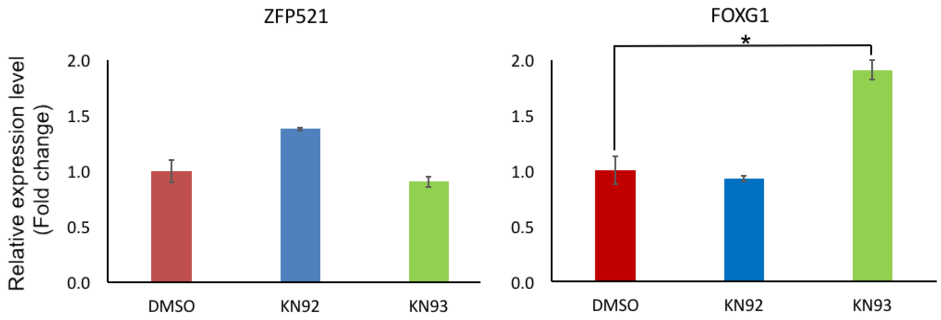

ZFP521
FOXG1
Relative expression level
(Fold change)
2.0
1.5
1.0
0.5
0.0
DMSO
KN92
KN93
*

Suppl. Figure 4. qRT-PCR analysis of *ZFP521* and *FOXG1* in DMSO-, KN92- and KN93-treated neural differentiating cells at day 7.

*$p < 0.05$ in comparison with the value from DMSO group.

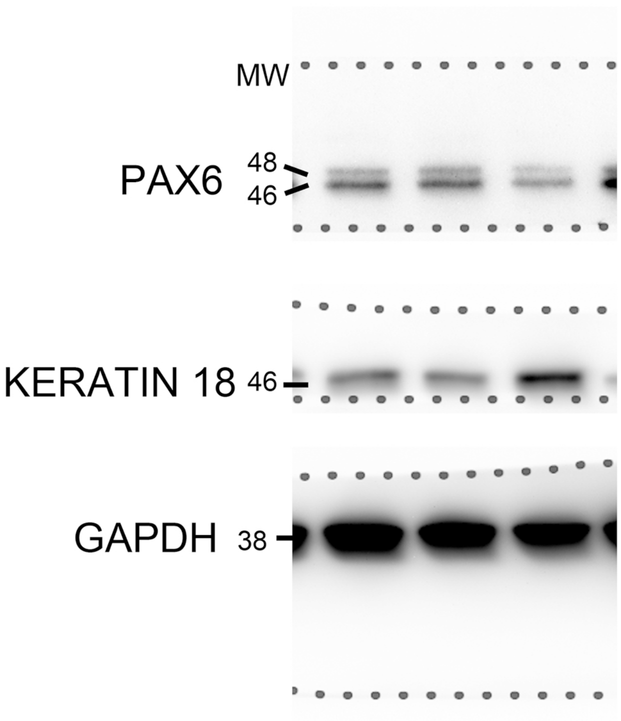
MW
PAX6
48
46
KERATIN 18
46
GAPDH
38

Suppl. Figure 5: Western blot images shown in Figure 5.

PAX6 antibody produced two bands at 46 and 48 kDa; whereas KERATIN18 antibody produced a band at 46 kDa**.**

Suppl. Table 1. Gene expression changes of mesodermal and endodermal layer markers of differentiating ESCs compared to ESCs

Gene symbol	Day 6	Day 10	Gene symbol	Day 6	Day 10
**Mesoderm**			**Endoderm**		
CD4	1.0	1.1	GATA1	1.0	1.0
GATA2	1.4	-1.1	SOX17	1.2	-1.1
RUNX1	-1.1	1.0	AFP	-2.5	-1.5
T	1.4	1.4	SOX7	1.7	-1.4

A positive value means the fold change of upregulation of the gene at Day 6 or 10 compared to ESCs (Day 0). A negative value means the fold change of downregulation of the gene at Day 6 or 10 compared to ESCs (Day 0).

Suppl. Table 2. Transcription factors that are downregulated and upregulated at day 6 of differentiation compared to ESCs

	log_2 value			
**Genes**	**Day 0**	**Day 6**	**Fold change**	**Known function**
**Downregulated Genes**				
**NANOG**	**11.18**	**8.35**	**-7.1**	**Self-renewal/Pluripotency**
**TGFB1I1**	**6.72**	**4.24**	**-5.6**	**Male sexual differentiation**
**NFIB**	**6.07**	**3.92**	**-4.4**	**N/D**
**HEY2**	**8.41**	**6.33**	**-4.3**	**Cardiovascular development**
**BHLHE40**	**6.07**	**4.13**	**-3.8**	**Control of circadian rhythm and chondrogenesis**
**BCL11A**	**7.77**	**5.87**	**-3.7**	**Hematopoietic cell differentiation**
**BCL11B**	**5.94**	**4.05**	**-3.7**	**Hematopoietic cell differentiation**
**ETS1**	**8.79**	**6.91**	**-3.7**	**Cell senescence, death, and tumorigenesis**
**DPPA2**	**7.43**	**5.57**	**-3.6**	**Pluripotency**
**TSHZ3**	**6.12**	**4.38**	**-3.3**	**Development of neurons involved in both respiratory rhythm and airflow control**
**FOXD3**	**6.79**	**5.1**	**-3.2**	**Self-renewal/Pluripotency**
**TXNIP**	**8.93**	**7.39**	**-2.9**	**Maturation of natural killer cells**
**TRNP1**	**8.77**	**7.23**	**-2.9**	**Regulates neural stem cells proliferation**
**EGR1**	**8.55**	**7.03**	**-2.9**	**Differentitation and mitogenesis**
**JAZF1**	**7.19**	**5.71**	**-2.8**	**Endometrial stromal tumors**
**BNC2**	**6.93**	**5.5**	**-2.7**	**Skin keratinocytes**
**FOXO1**	**9.42**	**8.02**	**-2.6**	**Metabolic homeostasis**
**POU5F1P3**	**11.09**	**9.7**	**-2.6**	**Pseudogene of POU5F1**
**MYC**	**9.19**	**7.88**	**-2.5**	**Cell cycle/ reprogramming**
**COMMD7**	**6.44**	**5.21**	**-2.4**	**N/D**
**POU5F1P4**	**10.99**	**9.84**	**-2.2**	**Pseudogene of POU5F1**
**ZNF398**	**9.23**	**8.08**	**-2.2**	**N/D**
**ID1**	**11.92**	**10.81**	**-2.2**	**Cell growth, senescence, and differentiation**
**DPPA4**	**9.51**	**8.41**	**-2.2**	**Pluripotency**
**TRPS1**	**5.12**	**4.02**	**-2.1**	**Regulates chondrocyte proliferation and differentiation**
**ZNF589**	**6.5**	**5.42**	**-2.1**	**Hematopoietic stem/progenitor cell differentiation**
**CDCA7L**	**10**	**8.93**	**-2.1**	**Apoptosis and proliferation**
**POU5F1**	**11.47**	**10.43**	**-2.1**	**Self-renewal/Pluripotency**
**POU5F1B**	**11.45**	**10.44**	**-2.0**	**Pseudogene of POU5F1**
**WWTR1**	**6.1**	**5.1**	**-2.0**	**Regulates embryonic stem-cell self-renewal**
**Upregulated Genes**				
**SIX3**	**4.36**	**10.51**	**70.87**	**Forebrain development**
**LHX2**	**3.28**	**9.35**	**67.5**	**Neural induction/ brain development**
**PAX6**	**3.03**	**8.6**	**47.53**	**Neural induction/ brain development**
**EPAS1**	**4.6**	**9.58**	**31.5**	**Development of blood vessels and tubular system of lung**

Suppl. Table 2. Cont.

Genes	log2 value Day 0	Day 6	Fold change	Known function
TFAP2B	3.42	7.53	17.2	Face and limb development and terminal differentiation and function of renal tubular epithelia
TFAP2A	5.42	9.25	14.3	early morphogenesis of the lens vesicle
DACH1	4.25	7.81	11.7	Regulation of organogenesis
LEF1	4.69	8.15	11.0	Hair cell differentiation and follicle morphogenesis
MEIS2	5.18	8.62	10.8	Eye development
ZEB2	3.61	7.01	10.5	Neural induction
SP8	5.23	8.63	10.5	Limb development
ISL1	4.78	8.17	10.5	Development of pancreatic cell lineages and may also be required for motor neuron generation
NR2F1	3.89	7.04	8.9	N/D
GATA3	2.96	6.03	8.4	T-cell development and endothelial cell biology
FOXC1	3.66	6.73	8.4	Cell viability and resistance to oxidative stress in the eye
ZNF521	6.23	9.14	7.5	Neural induction
IRX1	5.09	7.97	7.3	Tumor suppressor in gastric and head and neck cancers
NR2F2	5.39	8.24	7.2	N/D
DLX5	4.65	7.44	6.9	Osteoblast differentiation
HOXA1	3.89	6.66	6.9	Maintenance and/or generation of hindbrain segments
ZNF503	3.92	6.65	6.6	N/D
PAX3	3.9	6.51	6.1	Neural development and myogenesis
LMO3	4.92	7.43	5.7	N/D
SOX5	5.14	7.55	5.3	Regulation of embryonic development and in the determination of the cell fate
MSX2	4.25	6.65	5.3	Craniofacial morphogenesis
HESX1	8.32	10.7	5.2	Developing forebrain and pituitary gland
SOX9	5.09	7.42	5.0	Chondrocyte differentiation
MEIS1	3.99	6.31	5.0	Hematopoiesis, megakaryocyte lineage development and vascular patterning
IKZF2	4.97	7.28	5.0	Regulation of lymphocyte development
MAF	5.91	8.22	5.0	Embryonic lens fiber cell development

The top 30 transcription factors that have ≥ 2 fold change of downregulation and upregulation with $p < 0.05$ in a descending order are listed. The log2 value is the average of the absolute value of the probe. The known function of each gene is provided manually by authors according to literature, GeneCards Summary and/or UniProtKB/Swiss-Prot. N/D means the function of gene is not well-determined yet.

Suppl. Table 3. Transcription factors that are downregulated and upregulated at day 10 of differentiation compared to ESCs

	log_2 value			
**Genes**	**Day 0**	**Day 10**	**Fold change**	**Known function**
**Downregulated Genes**				
NANOG	11.18	5.26	-60.6	See Suppl. Table 2
BCL11A	8.93	4.1	-28.5	See Suppl. Table 2
POU5F1P3	11.09	6.54	-23.4	See Suppl. Table 2
POU5F1P4	10.99	6.47	-23.0	See Suppl. Table 2
ZFP42	8.12	3.93	-18.3	Reprogramming of X-chromosome inactivation
KLF4	8.32	4.41	-15.1	Pluripotency
POU5F1	11.47	7.56	-15.0	See Suppl. Table 2
POU5F1B	11.45	7.62	-14.3	See Suppl. Table 2
FOXH1	9.61	5.8	-14.1	N/D
HEY2	8.41	4.9	-11.5	See Suppl. Table 2
TRNP1	8.77	5.58	-9.1	See Suppl. Table 2
ZSCAN10	8.36	5.17	-9.1	Pluripotency
RUNX1T1	7.71	4.56	-8.9	Myeloid translocation gene family
ZNF165	7.04	4.01	-8.2	N/D
FOXO1	9.42	6.55	-7.3	See Suppl. Table 2
BNC2	6.67	3.92	-6.8	See Suppl. Table 2
OVOL2	7	4.3	-6.5	Mesenchymal to Epithelial Transition
NFIB	6.07	3.47	-6.1	N/D
TFAP2C	8.45	5.87	-6.0	Development of the eyes, face, body wall, limbs, and neural tube
FOXD3	6.79	4.23	-5.9	See Suppl. Table 2
MACC1	5.49	2.94	-5.9	Cellular growth, epithelial-mesenchymal transition, angiogenesis, cell motility, invasiveness, and metastasis
PRDM14	8.01	5.47	-5.8	Pluripotency
ETS1	8.79	6.35	-5.4	See Suppl. Table 2
DPPA2	7.43	5.05	-5.2	See Suppl. Table 2
ZBTB3	7.46	5.13	-5.0	N/D
JAZF1	6.69	4.53	-4.5	Endometrial stromal tumors
BCL11B	5.94	3.81	-4.4	See Suppl. Table 2
NFE2L3	7.91	5.79	-4.3	N/D
ZNF589	6.5	4.39	-4.3	Hematopoietic stem/progenitor cell differentiation
HHEX	5.66	3.58	-4.2	Hematopoietic differentiation
**Upregulated Genes**				
LHX2	3.28	11.43	283.8	See Suppl. Table 2
PAX6	3.03	10.05	129.7	See Suppl. Table 2
SIX3	4.36	10.77	85.2	See Suppl. Table 2
FOXG1	6.37	11.91	46.8	Development of the telencephalon

Suppl.Table 3. Cont.

Genes	log₂ value Day 0	Day 10	Fold change	Known function
DACH1	4.25	9.66	42.3	See Suppl. Table 2
NR2F1	3.89	9.08	36.4	N/D
SOX1	4.28	9.34	33.2	Brain and lens development
MEIS2	5.18	10.23	33.1	See Suppl. Table 2
SP8	5.23	10.2	31.3	See Suppl. Table 2
ZEB1	3.62	8.39	27.4	Epithelial-mesenchymal transition
HES5	5.84	10.53	25.9	Neurogenesis negative regulator
ZBTB16	4.07	8.66	24.2	Myeloid maturation and development and/or maintenance of other differentiated tissues
ZIC1	4.7	9.28	24.0	See Suppl. Table 2
EYA4	3.57	7.92	20.4	Eye development
SOX9	5.09	9.33	18.9	See Suppl. Table 2
MEIS1	3.99	8.12	17.6	See Suppl. Table 2
SOX5	5.14	9.25	17.3	See Suppl. Table 2
LMO3	4.92	8.98	16.7	N/D
SIX6	3.94	7.9	15.6	Eye development
ZNF503	3.92	7.75	14.3	N/D
NR2F2	5.39	9.08	12.9	N/D
SOX6	3.45	7.12	12.7	Development of the central nervous system, chondrogenesis and maintenance of cardiac and skeletal muscle cells
ZNF521	6.23	9.73	11.3	See Suppl. Table 2
PRDM16	3.37	6.69	10.0	Differentiation of brown adipose tissue
ZFHX4	4.79	7.95	9.0	Neural and muscle differentiation
EPAS1	4.6	7.72	8.7	See Suppl. Table 2
ZEB2	3.04	6.03	7.9	See Suppl. Table 2
FEZF2	3.94	6.9	7.8	Specification of corticospinal motor neurons and other subcerebral projection neurons
IKZF2	4.97	7.91	7.7	See Suppl. Table 2
LEF1	4.69	7.58	7.4	See Suppl. Table 2

The top 30 transcription factors that have ≥ 2 fold change of downreglation and upregulation with $p < 0.05$ in a descending order are listed. The log2 value is the average of the absolute value of the probe. The known function of each gene is provided manually by authors according to GeneCards Summary and/or UniProtKB/Swiss-Prot. N/D means the function of gene is not well-determined yet.

Suppl. Table 4. Primers for qRT-PCR

Gene	Forward	Reverse
GAPDH	gcaccgtcaaggctgagaac	agggatctcgctcctggaa
POU5F1	cagtgcccgaaacccacac	ggagacccagcagcctcaaa
PAX6	tctttgcttgggaaatccg	ctgcccgttcaacatccttag
KR18	atgcgccagtctgtggag	cctgagatttgggggcatc
BCL11A	cgccagaggatgacgattgtt	ccaggcgtggggattagag
BCL11B	tccagctacatttgcacaaca	gctccaggtagatgcggaag
ZBTB16	gagatcctcttccaccgcaat	ccgcatacagcaggtcatc
SP8	acttctagggcgtggtgctt	cagaggagtcgatcccaacc
TFAP2B	ttcctcccaaatcggtgactt	cgccggtgttgacagacat
EPAS1	cccatgtctccaccttcaag	ggcttgctcttcatactccag
LEF1	agacaagcacaaacctctcag	tcattatgtacccggaataactcg
HEY2	aaggcgtcgggatcggataa	agagcgtgtgcgtcaaagtag
DNMT3B	cccagctcttaccttaccatcg	ggtcccctattccaaactcct
TET2	ggctacaaagctccagaatgg	aagagtgccacttggtgtctc
KAT2B	aggaaaacctgtggttgaagg	cagtcttcgttgagatggtgc
SIRT1	tgtgtcataggttaggtggtga	agccaattctttttgtgttcgtg
HDAC6	gagggagaactccgtgtccta	aatagccatccataagactgtgc
SETBP1	gtccacctgagatcaagatc	taagcctgtggctgaaatcc
SETD7	ggccagggagtttacacttac	ctcatcagggtacacataggcta
SMYD2	ctccaagcatctcggattccc	tgcaacatcaggaaatatcgctg
KDM4C	cgaggtggaaagtcctctgaa	gggctcctttagactccatgtat
WNT5B	cgcttcgccaaggagtttg	tgccatcttatacacagccct
ZFP521	caagcgaaaccgagatccctc	tcttccggcctcttcttacaat
FOXG1	cgtccaccatatagttccatga	tgactgctttgccatttcattc
